# Coronary Endothelial Dysfunction and Vasomotor Dysregulation in Myocardial Bridging

**DOI:** 10.3390/jcdd12020054

**Published:** 2025-02-02

**Authors:** Takumi Toya

**Affiliations:** 1 Division of Cardiology, National Defense Medical College, Tokorozawa 359-8513, Japan; tyt0725@gmail.com; 2 Department of Cardiovascular Medicine, NHO Tokyo Medical Center, Tokyo 152-8902, Japan

**Keywords:** myocardial bridging, coronary endothelial dysfunction, coronary spasm, coronary microvascular dysfunction

## Abstract

Myocardial bridging (MB), a congenital variant where a coronary artery segment is tunneled within the myocardium, is increasingly recognized as a contributor to coronary endothelial and vasomotor dysfunction. Beyond the hallmark systolic compression observed on angiography, MB disrupts endothelial integrity, impairs the release of vasoactive substances, and induces vasomotor abnormalities. These effects exacerbate ischemic symptoms and predispose to atherosclerosis in the proximal segment, particularly in conditions such as ischemia/myocardial infarction with nonobstructive coronary arteries. Recent studies underscore MB’s association with coronary vasospasm, microvascular endothelial dysfunction, and adverse cardiovascular outcomes, including sudden cardiac death. These findings highlight the interplay between MB’s structural anomalies and functional impairments, with factors such as the bridge’s length, depth, and orientation influencing its hemodynamic significance. Advances in imaging and coronary physiology assessment, including acetylcholine testing and stress diastolic fractional flow reserve/iFR/RFR, have enhanced diagnostic precision. This review explores the multifaceted impact of MB on coronary physiology, emphasizing its role in endothelial dysfunction and vasomotor regulation. Recognizing MB’s contribution to cardiovascular disease is essential for accurate diagnosis and tailored management strategies aimed at mitigating ischemic risk and improving patient outcomes.

## 1. Introduction

Myocardial bridging (MB), a congenital anomaly where a segment of a coronary artery is tunneled within the myocardium, has traditionally been regarded as a benign variant. However, growing evidence highlights its significant contribution to endothelial dysfunction and vasomotor dysregulation. These pathophysiological changes extend beyond the hallmark systolic compression of the tunneled segment, affecting both epicardial and microvascular function. Endothelial dysfunction due to MB impairs the release of vasoactive substances, leading to abnormal vasomotor responses and heightened susceptibility to coronary vasospasm and ischemia. Furthermore, these hemodynamic disturbances increase the risk of ischemic symptoms, even in the absence of obstructive coronary artery disease. Emerging insights also suggest a link between MB and coronary microvascular endothelial dysfunction, compounding its clinical significance.

This narrative review aims to provide a comprehensive analysis of the multifaceted impact of MB on coronary endothelial function and vasomotor regulation, with a particular focus on its contribution to ischemic pathophysiology and adverse cardiovascular outcomes, including sudden cardiac death. By synthesizing recent advances in the field, this review seeks to elucidate the complex interplay between MB and endothelial and vasomotor dysfunction, thereby offering a foundation for developing tailored therapeutic approaches for patients with MB.

## 2. Prevalence and Hemodynamic Significance of MB

MB is a common anatomical variant, with prevalence estimates varying significantly depending on evaluation methods and the characteristics of the target population; however, MB is likely present in approximately one-third of individuals [1]. Autopsy studies consistently report MB in 33–42% of individuals [2,3,4], while detection rates using coronary angiography range from 0.5 to 16%, rising to 40% with provocation testing using nitroglycerin or dobutamine [5,6,7,8,9]. Coronary computed tomography angiography is more sensitive than coronary angiography, detecting MB in up to 58% of cases [5,6]. Conventional angiography identifies dynamic systolic compression—a hallmark feature of MB—in only 13.3% of patients with MB detected by coronary computed tomography angiography [6]. This variability in prevalence reflects differences in target populations, imaging modalities, and inclusion criteria, including distinctions between superficial and deep MB. The majority of MB (67–98%) is located in the left anterior descending artery, with the mid- and proximal left anterior descending artery being the most affected segments, whereas MB in the left circumflex or right coronary arteries are less common [10]. Notably, dynamic compression, the angiographic hallmark of MB, is almost exclusively observed in the left anterior descending artery [6]. Beyond systolic compression, MB has significant hemodynamic and pathophysiological consequences. The repetitive compression and decompression cycles disrupt endothelial integrity, impairing the release of vasoactive substances and leading to vasomotor dysfunction. These alterations limit coronary flow augmentation, increase the risk of atherosclerosis proximal to the bridged segment, and heighten ischemic symptomatology. Factors such as the length, depth, and orientation of the bridge, as well as myocardial fiber arrangement, surrounding tissue composition, coronary tone, and hemodynamic conditions, further influence the clinical impact of MB [9,11]. Recent advances in imaging and coronary physiology assessment have deepened our understanding of MB’s anatomy and pathophysiology, emphasizing the need for a comprehensive diagnostic approach. These insights, along with their clinical implications, will be further explored in the subsequent sections.

## 3. Epicardial Endothelial Dysfunction and Myocardial Bridging

The primary functional consequence of MB is the compression of the coronary artery lumen during systole, extending into diastole [7,12]. This subsequently leads to hemodynamic alterations at the MB site, consistent with the conceptual model of a high-pressure and high-shear stress chamber within MB [12]. While a moderate level of shear stress is generally considered beneficial for vascular health, the extremes of shear stress, whether high or low, can potentially disrupt endothelial integrity and normal function [13,14]. Furthermore, it is worth highlighting that elevated intravascular pressure has also been associated with endothelial dysfunction [15,16,17,18].

A case–control study compared endothelial-dependent and endothelial-independent vasomotor function between 29 patients with MB and 58 propensity score-matched control patients without MB. The endothelium-independent vasomotor function, as determined by coronary flow velocity reserve after intracoronary adenosine injection, did not differ between the two groups. In contrast, the endothelium-dependent vasomotor function was explicitly impaired at the site of MB in comparison to both proximal and distal coronary segments and to the control group. The wall shear rate was significantly elevated in the MB site, underscoring the complex relationship between structural and functional aspects of this condition [19].

Consistent with the study above, in several cohorts characterized by ischemia with nonobstructive coronary artery disease (INOCA), coronary vasospasm was significantly more frequent in patients with MB compared to those without this anatomic variant. In a cohort of 114 Japanese patients who underwent coronary angiography and acetylcholine provocation testing to assess chest pain, individuals with MB displayed a significantly higher incidence of coronary spasms than their counterparts without MB (73% vs. 40%). Furthermore, MB emerged as an independent predictor of coronary spasm, with an odds ratio of 3.5 [20]. Similarly, a study conducted in a Korean population reported a significantly higher prevalence of coronary spasm in those with MB compared to those without (77% vs. 16%), supporting these findings. Focal spasms confined primarily to the bridged segment were observed in a substantial proportion (64%) of patients with MB, while such focal spasms were rarely detectable (8%) in those without MB [21]. Furthermore, within a cohort comprising 392 patients who underwent acetylcholine provocation testing, patients with MB (36%) exhibited a greater prevalence of provoked coronary spasms than those without MB (59% vs. 43%). It is worth noting that the anatomic severity of MB, as quantified by the length of the tunneled segment and the extent of systolic compression, positively correlated with the likelihood of coronary spasms [22]. A recent study also revealed that the anatomical severity of MB gauged by MB muscle index on coronary computed tomography angiography was independently associated with epicardial endothelial dysfunction (length [mm]*coverage grade) [23]. Significantly, among patients with MB, those experiencing coronary spasms were more predisposed to recurrent angina when compared to those without coronary spasms, underscoring the pivotal role of coronary spasms in precipitating ischemic symptoms in patients with MB [24].

Previous studies have consistently reported that coronary spasms can cause myocardial injury even in individuals without obstructive coronary artery disease. Both epicardial and microvascular coronary spasms have been established as important triggers of myocardial infarction and nonobstructive coronary artery disease (MINOCA) [25,26,27,28]. However, the precise role of MB in the pathogenesis of MINOCA remains ambiguous. Recent findings from a prospective study involving 310 patients with nonobstructive coronary artery disease undergoing acetylcholine provocation testing, 54.5% of whom present with INOCA and 45.5% with MINOCA, support the notion that MB may induce not only ischemic symptoms but also ischemic myocardial injury by inducing endothelial dysfunction, ultimately leading to the development of coronary spasms. In this study, MB emerged as an independent predictor of provocative coronary spasms and subsequent major adverse cardiovascular events (MACE; cardiac death, nonfatal myocardial infarction, and rehospitalization due to unstable angina). In particular, patients harboring MB with provocative coronary spasms were identified as having the highest risk of MACE [29]. These findings underscore the potential clinical significance of MB in the complex interplay between endothelial dysfunction and adverse cardiovascular outcomes.

## 4. Coronary Microvascular Endothelial Dysfunction and Myocardia Bridging

MB has been implicated as a possible contributor to the development of coronary microvascular endothelial dysfunction (CMED), but the exact mechanism remains to be elucidated. In a cohort of 1469 INOCA patients who underwent coronary angiography with coronary vasomotor function testing, a significant proportion (14.2%) had MB in the middle and distal portion of the left anterior descending artery [30]. Notably, patients with MB had a higher propensity for both epicardial endothelial dysfunction and CMED than those without MB. Interestingly, the study revealed the demographic characteristic that patients with MB were younger and had fewer traditional cardiovascular risk factors, suggesting a plausible scenario in which MB may cause ischemic symptoms beyond those attributable to conventional cardiovascular risk factors. The effect of MB on the development of CMED was more pronounced in those aged < 50 years than in those aged > 50 years [30]. This discrepancy raises the possibility that vasoactive substances, particularly endothelin-1, may be a plausible mediator linking epicardial MB and downstream microvascular endothelial dysfunction. MB has been shown to exert a modulatory effect on regional concentrations of endothelin-1, and the bioavailability of this vasoactive substance is known to exhibit age-related changes [31,32]. These findings highlight the potential for age-related changes in vasoactive substances to mediate the interaction between MB and the development of CMED, thereby enhancing our understanding of the complex pathophysiological linkages that underlie these phenomena.

## 5. Sudden Death and Myocardial Bridging

As discussed in the previous sections, MB is an independent risk factor for coronary spasms and is associated with an elevated risk of MACE. Notably, patients with MB who test positive for acetylcholine provocation exhibit the highest risk of a composite of death, myocardial infarction, and hospitalization for unstable angina [29]. In a study involving patients undergoing implantable cardioverter defibrillator implantation (N = 23 for primary prevention, N = 117 for secondary prevention), MB and coronary spasm independently predicted life-threatening ventricular arrhythmias, even after adjusting for other risk factors. Longer MB was particularly associated with these arrhythmias, consistent with prior findings that MB length predisposes patients to coronary spasms [22,33]. Longer MB is also linked to hemodynamically significant MB, defined as a diastolic fractional flow reserve ≤0.76, and is associated with myocardial fibrosis and interstitial edema in autopsy studies of sudden cardiac death [34,35,36]. Beyond functional abnormalities, MB contributes to accelerated coronary atherosclerosis in the proximal segment, predisposing patients to myocardial infarction [37]. This phenomenon is likely due to low and oscillatory wall shear stress at the proximal entrance of the MB segment [19,38,39,40]. Even in heart transplant recipients, MB in transplanted hearts has emerged as a risk factor for death and re-transplantation. MB facilitates the progression of allograft vasculopathy in the proximal segment, with arterial compression correlating with increased plaque burden [41]. Taken together, these studies suggest that longer MB is associated with coronary spasms or a hemodynamically significant decrease in coronary perfusion due to compression or coronary atherogenesis, potentially leading to myocardial ischemic injury-induced ventricular arrhythmia and sudden cardiac death. Other studies also point to the link between MB and ventricular arrhythmia and sudden cardiac death [42,43,44]. MB is associated with significant increases in exercise-induced premature ventricular tachycardia and non-sustained ventricular tachycardia, as well as increases in QT dispersion and repolarization abnormalities, all of which are known to be associated with ventricular arrhythmia and sudden cardiac death [41]. Given the strong association between MB and adverse cardiovascular outcomes, particularly life-threatening arrhythmias and sudden cardiac death, a comprehensive diagnostic approach is warranted.

## 6. Diagnostic Procedure Unveiling Coronary Endothelial Dysfunction and Significant Myocardial Bridging

Invasive diagnostic procedures for evaluating MB can be categorized into two main approaches. The first focuses on assessing the hemodynamic significance of coronary compression within the tunneled segment. The second evaluates alterations in epicardial and microvascular endothelial function using acetylcholine, which often manifests as coronary spasms or coronary vasodilatory dysfunction associated with MB.

While fractional flow reserve (FFR) is the gold standard for evaluating the significance of obstructive coronary artery disease, it is not reliable for assessing the physiological impact of MB. The systolic compression caused by MB leads to an overshoot in distal coronary pressure, potentially masking significant flow restriction and yielding falsely negative FFR results. Additionally, delayed decompression of the bridged segment—attributable to prolonged ventricular relaxation—can impair diastolic hyperemic flow, during which approximately 85% of coronary blood flow occurs. This effect is further exacerbated by tachycardia and increased sympathetic drive during exercise or emotional stress, both of which reduce diastolic perfusion time, thereby reducing coronary flow and myocardial perfusion. These diastolic flow impairments underlie the pathophysiological consequences associated with MB [45]. Consequently, diastolic FFR (dFFR) or instantaneous wave-free ratio (iFR), focusing specifically on the diastolic phase, is theoretically more accurate in assessing the true physiological significance of MB compared to conventional mean pressure FFR [46]. Resting full-cycle ratio (RFR), calculated as the minimum value during systole and diastole, may also better reflect MB’s hemodynamic significance [47]. Dobutamine stress testing, by amplifying systolic compression and heart rate, is recommended for uncovering physiologically significant MB. A dobutamine stress dFFR/iFR/RFR ≤ 0.76 is considered a marker of hemodynamic significance, although further validation is needed [46,48].

Coronary endothelial dysfunction is characterized by an abnormal vasomotor response to a vasodilating agent, such as acetylcholine, and manifests as coronary spasms (excessive vasoconstriction) or impaired vasodilation [49,50,51]. The Coronary Vasomotor Disorders International Study Group (COVADIS) defines epicardial spasms as ≥90% stenosis of the coronary artery diameter with ischemic symptoms and ECG changes following intracoronary acetylcholine administration [52]. Microvascular spasms are diagnosed when ischemic symptoms and ECG changes occur without significant epicardial spasm [53]. Alternative criteria for microvascular spasm include an inverted lactate concentration gradient between coronary arteries and the coronary sinus after acetylcholine infusion [54,55].

Epicardial endothelial dysfunction may also be indicated by constriction of the coronary artery diameter (>20% reduction) during acetylcholine infusion, even in the absence of coronary spasms [50,56]. Conversely, coronary microvascular endothelial dysfunction is defined by inadequate coronary blood flow augmentation (<50%) during acetylcholine infusion [57,58,59,60]. Variability in acetylcholine dosing and administration across facilities complicates interpretation, as different protocols may yield inconsistent results [61,62]. However, accurate assessment of coronary vasomotor dysfunction, particularly coronary spasms, is crucial for guiding therapy and improving patient outcomes. Tailored therapeutic strategies should consider the presence or absence of coronary spasms to optimize the management and long-term prognosis of patients with MB.

## 7. Therapeutic Considerations for Patients with Coronary Endothelial Dysfunction and Myocardial Bridging

Pharmacologic therapy remains the cornerstone of treatment for most patients with symptomatic MB despite the lack of randomized clinical trial data. Beta-blockers are generally considered first-line agents due to their negative chronotropic and inotropic effects, which decrease heart rate, prolong diastolic filling time, and reduce arterial compression, alleviating hemodynamic disturbances caused by MB [9,63,64]. However, for patients with concomitant coronary vasospasms, beta-blockers may potentially exacerbate coronary spasms [65,66]. Non-dihydropyridine calcium-channel blockers serve as an alternative, especially in patients with contraindications to beta-blockers, such as bronchospasm, and their additional vasodilatory effects may benefit patients with coexisting coronary spasm [40,67]. Head-to-head comparison of beta-blockers and calcium-channel blockers is not available targeting the patients harboring MB with coronary spasms. Ivabradine, an off-label option, may be considered as a second-line agent for heart rate reduction in patients intolerant to or inadequately treated with beta-blockers or calcium-channel blockers [68]. Importantly, nitrates are contraindicated in MB, as they can intensify systolic compression of the bridged segment, exacerbate retrograde flow, and lower the ischemic threshold, though their antispasmodic properties might have limited utility in cases of concomitant coronary spasms [8,40,68,69]. Furthermore, MB is associated with an increased risk of atherosclerosis, particularly proximal to the bridged segment [45,70]. Aggressive cardiovascular risk factor modification is recommended, and antiplatelet therapy can be considered in the presence of detected atherosclerosis. Non-invasive imaging modalities, particularly coronary computed tomography angiography, play a pivotal role in identifying subclinical atherosclerosis and assessing plaque vulnerability, thereby guiding therapeutic strategies [40,71]. Recent advances in imaging technologies, such as photon-counting computed tomography, have enabled more detailed assessments of coronary arteries with superior spatial resolution, enhanced contrast, and reduced artifacts. In addition to anatomical evaluation, computed tomography-derived fractional flow reserve (CT-FFR) has emerged as a valuable tool for assessing the physiological significance of obstructive stenosis in patients with MB [72]. However, a cautious interpretation of CT-FFR findings is necessary due to potential artifacts [73]. CT-FFR may also predict the progression of proximal coronary atherosclerosis related to MB. Lower CT-FFR values and higher CT-FFR gradients between segments proximal and distal to the MB are associated with plaque progression in proximal to MB, thereby identifying patients who may benefit from intensified pharmacotherapy and closer follow-up [74].

When significant obstructive coronary lesions develop proximal to MB, percutaneous coronary intervention (PCI) can be considered. However, stent deployment should be approached with caution, as evidence indicates that stents extending into the bridged segment are associated with a significantly higher target vessel revascularization rate (29%) compared to stents ending proximal to the MB (3%) [75]. Also, PCI for significant MB should be reserved for patients with refractory symptoms after maximizing pharmacotherapy, given ongoing concerns about its long-term efficacy. In-stent restenosis rates remain substantial, with rates of up to 75% for bare-metal stents and 25% for early-generation drug-eluting stents [76]. Although newer-generation drug-eluting stents with enhanced radial strength may offer better resilience against cyclic systolic compression, data on their efficacy in MB-related PCI remains limited. A key concern is whether stenting effectively addresses the underlying endothelial and vasomotor dysfunction associated with MB, particularly in arteries prone to spasmodic activity. These issues underscore the need for further investigation to elucidate the benefits and risks of PCI in this context and to optimize treatment strategies for patients with MB.

## 8. Summary and Future Perspectives

MB is more than an anatomical anomaly; it is a dynamic contributor to coronary endothelial dysfunction and vasomotor dysregulation. The repetitive compression and decompression cycles inherent to MB disrupt endothelial integrity, impair vasodilatory capacity, and predispose to coronary spasm and microvascular dysfunction. These pathophysiological effects amplify ischemic risk, even in patients without significant coronary artery obstruction. Recognition of the endothelial and vasomotor dysfunction associated with MB is critical for accurate diagnosis and effective management. Advanced imaging modalities and coronary function testing have illuminated the underlying mechanisms of MB-related dysfunction, paving the way for targeted therapies. Future research should focus on unraveling the precise molecular and hemodynamic pathways linking MB to cardiovascular events and developing interventions that address both its structural and functional consequences.
